# Mechanisms of recognition and binding of α-TTP to the plasma membrane by multi-scale molecular dynamics simulations

**DOI:** 10.3389/fmolb.2015.00036

**Published:** 2015-07-01

**Authors:** Christos Lamprakis, Achim Stocker, Michele Cascella

**Affiliations:** ^1^Department of Chemistry and Biochemistry, University of BernBern, Switzerland; ^2^Department of Chemistry, Centre for Theoretical and Computational Chemistry, University of OsloOslo, Norway

**Keywords:** sec14-like, vitamin E, PIP, coarse grained, lipid exchange

## Abstract

We used multiple sets of simulations both at the atomistic and coarse-grained level of resolution to investigate interaction and binding of α-tochoperol transfer protein (α-TTP) to phosphatidylinositol phosphate lipids (PIPs). Our calculations indicate that enrichment of membranes with such lipids facilitate membrane anchoring. Atomistic models suggest that PIP can be incorporated into the binding cavity of α-TTP and therefore confirm that such protein can work as lipid exchanger between the endosome and the plasma membrane. Comparison of the atomistic models of the α-TTP-PIPs complex with membrane-bound α-TTP revealed different roles for the various basic residues composing the basic patch that is key for the protein/ligand interaction. Such residues are of critical importance as several point mutations at their position lead to severe forms of ataxia with vitamin E deficiency (AVED) phenotypes. Specifically, R221 is main residue responsible for the stabilization of the complex. R68 and R192 exchange strong interactions in the protein or in the membrane complex only, suggesting that the two residues alternate contact formation, thus facilitating lipid flipping from the membrane into the protein cavity during the lipid exchange process. Finally, R59 shows weaker interactions with PIPs anyway with a clear preference for specific phosphorylation positions, hinting a role in early membrane selectivity for the protein. Altogether, our simulations reveal significant aspects at the atomistic scale of interactions of α-TTP with the plasma membrane and with PIP, providing clarifications on the mechanism of intracellular vitamin E trafficking and helping establishing the role of key residue for the functionality of α-TTP.

## 1. Introduction

The dietary balance of all superior animals must include periodic assimilation of vitamin E, one of the most important antioxidants capable of quenching singlet oxygen, protecting from peroxidative damage, and of capturing other radical species (Tappel, 1962; Herrera and Barbas, 2001; Packer et al., 2001). Of all the chemical species associated to the generic *vitamin E* name, superior animals typically retain only RRR-α-tocopherol (α-Tol hereafter). This process occurs in multiple steps: initially, all vitamin isoforms are absorbed in the small intestine, delivered to the lymph, and eventually incorporated into early endosomal compartments of epatocytes (Traber and Sies, 1996; Yap et al., 2001). Upon maturation of the endosomes, α-Tol is selectively recognized and extracted from the endosomal membranes by α-tocopherol-transfer protein (α-TTP hereafter), a cytosolic transporter of ≈ 32 kDa weight belonging to the Sec14 like family (Meier et al., 2003; Min et al., 2003). α-TTP mobilizes the substrate, allowing its transfer into the inner leaflet of the plasma membrane, and eventually leading to its delivery into the blood-stream (Oram et al., 2001; Horiguchi et al., 2003). Missense mutations in α-TTP disrupt such a crucial step required for α-Tol delivery into the body, leading to severe degenerative disease called Ataxia with Vitamin E Deficiency (AVED) (Donato et al., 2010).

Despite such coarse picture is understood, the molecular details by which such processes occur are far from being elucidated. In particular, it is not clear how α-TTP recognizes and binds to the different cellular membranes, or how ligands are incorporated or released by the protein. Influence of environmental variables onto the process (like decrease of pH during endosomal maturation, membrane curvature, or presence of lipid rafts) are also at present not well understood.

In recent times, we identified the molecular means by which α-TTP selects α-Tol over other chemical variants by combining computational approaches to biochemical *in vitro* data. In particular, we showed how optimal binding of ligands into the large hydrophobic cavity of α-TTP is affected by slight chemical modifications at the tocopherol, and also how site-directed sequence modifications of the amino acids composing the binding pocket can efficiently modulate the selectivity of α-TTP, favoring other substrate isospecies (Helbling et al., 2012). The specificity had been studied in the past by measuring the dissociation constants of various ligands in α-TTP (Panagabko et al., 2003). Previous works had also studied the effect of specific AVED mutations in the affinity for α-Tol (Bromley et al., 2013), the effect of the membrane's phospholipid composition and curvature (Zhang et al., 2009) as well as the contribution of α-TTP's surface residues to membrane binding and ligand transfer (Zhang et al., 2011).

Protein dynamics at different membrane compartments may involve interaction with charged lipids. In particular, phosphatidylinositol phosphates (PIPs) have been long identified as key landmarks for peripheral proteins to distinguish between different organelles (Munro, 2002). Recently, it has been shown that α-TTP is able to bind directly to PIPs (Kono et al., 2013). Such a binding is consistent with similar interactions already seen in other members of the Sec14 family (Krugmann et al., 2002; Huynh et al., 2003; Merkulova et al., 2005; Katoh et al., 2009; Saari et al., 2009). The structure of such a complex has been resolved in the case of the yeast Sec14 homolog 1 (Schaaf et al., 2008).

Arai and coworkers (Kono et al., 2013) suggested that the transferring of α-Tol to the plasma membrane is coupled to the extraction of PIPs from this membrane, through a ligand-exchange mechanism between PIPs and α-Tol. Such mechanism includes the capturing of α-Tol from the late endosome outer leaflet and its transportation to the cytosolic facing leaflet of the plasma membrane (Horiguchi et al., 2003). Interestingly, three of the AVED mutations (R59W, R192H, and R221W) directly affect the charged cleft in the area that hinges the mobile gate. When these residues were mutated, α-TTP could no longer bind to negatively charged PIPs (Kono et al., 2013). In the same study, two crystal structures (PDB entries:3W67, 3W68) of the bound mouse α-TTP to two different phosphatidylinositol biphosphates (PIP_2_) molecules were produced (Kono et al., 2013). Such structures revealed the binding poses of the phosphatidylinositol 3,4-bisphosphate [PI(3,4)P_2_] and the phosphatidylinositol 4,5-bisphosphate [PI(4,5)P_2_], which are both present to the plasma membrane (Kono et al., 2013). The binding of both PIP_2_ involves direct salt-bridge formation between the negatively-charged phosphate groups and the positively-charged residues at the basic patch in the proximity of the ligand binding cavity. Unfortunately, in these complexes, the acyl chains were not fully resolved [especially in the case of PI(4,5)P_2_] and positioned parallel to and in between the mobile gate helix responsible for opening the binding cavity to the exterior of the protein and the corresponding protein interface. Also, the natural ligand α-Tol is still present in the binding pocket of the protein. Thus, the experimental structure most probably captured an intermediate state between the open and closed conformations of α-TTP, where the two ligand were not actually exchanged.

Apart from the molecular details of the interaction between α-TTP and the single PIP molecules, a more complete structural and dynamical model of such adduct in the presence of the complex plasma membrane would be also desirable. Studing this system from such a perspective, would reveal the importance of each of these different residues during the complicated procedure of membrane recognition binding and lipid exchange. An explanation of the AVED phenotype caused by the aforementioned mutations could be provided by studying the whole procedure and not relate their implications straight to the overlall structure/properties of the protein *per se* (Bromley et al., 2013).

In this study, we used both All Atom (AA) and Coarse Grained (CG) molecular dynamics (MD) simulations to investigate the interactions of α-TTP to different PIP_2_ and to the plasma membrane. A multi-scale simulation approach is used. First, the CG model of α-TTP-PIP_2_ lipid bilayer are used to identify the global behavior of α-TTP in the presence of a PIP_2_-rich membrane model; then, full atomistic models are used to refine the description of the molecular interactions and conformational dynamics responsible for the anchoring of α-TTP to a model of the plasma membrane. AA dynamics is also used to determine putative structures of the binding of PIP_2_ into the ligand cavity of α-TTP in the closed state of the protein, after exchange of the lipids.

## 2. Materials and methods

### 2.1. α-TTP-PIP_2_ complex

#### 2.1.1. System setup

We used the crystal structure of the mouse α-TTP in complex with α-Toc and PI(3,4)P_2_ lipids (pdb entry: 3W67) (Kono et al., 2013) as our initial starting model, because it has more fatty acid atoms resolved, compared to the one with PI(4,5)P_2_. After the removal of α-Toc, the missing atoms of the acyl chains were reconstructed as a random chain, in the area between helices α9 and α10, using the most frequent arachidonoyl (20:4 n-6) and stearoyl (18:0) stoichiometry (Tanaka et al., 2003). An acetyl group capped the protein's trimmed N-terminus to prevent the overestimation of its interaction with the highly negatively charged PIP_2_ head group. Protonation states were assigned by the PROPKA software at pH 7.0 (Li et al., 2005) and hydrogen atoms were added to the structure according to atomic valence. The AMBER FF99SB (Cornell et al., 1995a; Cheatham et al., 1999; Hornak et al., 2006) and the LIPID11 (Skjevik et al., 2012) force fields were used to parameterize the protein and acyl chains, respectively. The standard RESP procedure (Cornell et al., 1995a) was followed to assign atomic charges to the polar part of the PI(3,4)P_2_ lipid, matching ab initio calculations at the B3LYP (Stephens et al., 1994) level of theory with the 6-31G^**^ basis set. The latter calculations were performed with the GAUSSIAN03 software (Aaqvist, 1990; Frisch et al., 2004). Finally their atom types were determined from the LIPID11 (Skjevik et al., 2012) force field. Following the hydration with 14384 TIP3P (Jorgensen et al., 1983) water molecules, 3 Na^+^ (Aaqvist, 1990) atoms were added to neutralize the system resulting in a total system of 47,430 atoms in a box with dimensions 76.5 × 78 × 87Å^3^.

#### 2.1.2. Computational details

All simulations were performed with the NAMD package (Phillips et al., 2005). After 5000 steps of conjugate gradient minimization, the system was slowly heated from 0 to 300 K in the NVT ensemble over 60 ps of MD using a time-step of 2 fs, and a Langevin thermostat to control the temperature. The system was equilibrated for 0.5 ns in the NPT ensemble constraining the non water atoms with a harmonic constraint to allow the water to relax. Thereafter, the simulation was continued in the same ensemble for 250 ns. at a temperature of 300 K and pressure 1 bar controlled by the Langevin thermostat and the Nosé-Hoover Langevin barostat implemented in NAMD (Phillips et al., 2005). The Particle Mesh Ewald method (Essmann et al., 1995) was used to compute the full system periodic electrostatics, the van der Waals potential was computed using a cutoff of 12 Å. All bonds between hydrogen and any other atom were constrained to their equilibrium length using the SHAKE algorithm (Ryckaert et al., 1977).

The final model obtained for the α-TTP - PI(3,4)P_2_ complex was used as the starting point of α-TTP - PI(4,5)P_2_. The structure of the phosphorylated head-groups was obtained by molecular replacement after superposition of the α-TTP - PI(3,4)P_2_ structure coming from MD with the X-ray data available for α-TTP - PI(4,5)P_2_.

### 2.2. Model of α-TTP in complex with α-Tol at plasma membrane

#### 2.2.1. Coarse grained simulations

##### 2.2.1.1. System setup

Two systems were constructed in the Martini coarse-grained representation (Marrink et al., 2007; Monticelli et al., 2008). Both contained one α-TTP protein and a bilayer consisting of 270 1,2-Dioleoyl-sn-Glycero-3-Phosphoethanolamine (DOPC) and 180 1,2-Dioleoyl-sn-Glycero-3-Phosphoethanolamine (DOPE) lipid molecules keeping a ratio 3:2. Moreover, four PI(3,4)P_2_ molecules were added in the bilayer and 19 Na^+^ atoms to neutralize the system. The systems were hydrated by using 16815 molecules of the Martini polarisable water model (Yesylevskyy et al., 2010) to achieve a better screening of the electrostatic interactions, resulting in a box of 12.2 × 12.2 × 17.7 Å^3^. The difference in the two systems is the existence of one PI(3,4)P_2_ molecule loaded in the binding pocket of α-TTP. The Martini force field parameters (Lopez et al., 2009, 2013; de Jong et al., 2013) were used to describe the system. Moreover, the elastic network model (Periole et al., 2009) was used additionally in the case of the protein's topology to assist the preservation of its higher order structure. The CG model followed the Martini description. The latter has been validated in several examples (Stansfeld et al., 2009; van den Bogaart et al., 2011; Lumb and Sansom, 2013; Schmidt et al., 2013) in systems of proteins interacting with PIPs incorporated in membrane model systems.

##### 2.2.1.2. Computational details

Following 5000 steps of steepest descent minimization, the system was equilibrated in the NPT ensemble. For this purpose, thermal and pressure baths according to Berendsen et al. were used to couple temperature (300 K) and pressure (1atm) with 1.0 ps coupling constants in a semi-isotropic scheme. The van der Waals interactions were described by a Lennard-Jones potential that was shifted from 9 to 12 Å. The electrostatics decay to zero from 0 to 12 Å using a dielectric constant of 2.5. A time step of 15 ns was used to integrate the Newton equation of motion of the Leap-frog algorithm for a total time of 90 and 170 ns for the two systems. The MD simulations were performed using the Gromacs software (Berendsen et al., 1995; Lindahl et al., 2001; van der Spoel et al., 2005; Hess et al., 2008).

#### 2.2.2. All atom simulations

##### 2.2.2.1. System setup

The systems contained the α-TTP in complex with α-Toc interacting with a DOPC/DOPE membrane containing either a PI(4,5)P_2_ or a PI(3,4)P_2_ molecule. For the protein, α-Toc, PI(4,5)P_2_ and PI(3,4)P_2_ the same protocol as above was used. The membrane lipids were parameterized with the LIPID11 force field (Skjevik et al., 2012). A number of 6 Na^+^ atoms (Aaqvist, 1990; Frisch et al., 2004) and 18566 TIP3P water molecules (Jorgensen et al., 1983) were used to neutralize and solvate the system. Equilibration and production runs (200 ns each) were conducted following the same schemes as for the atomistic simulations for the α-TTP.

## 3. Results

### 3.1. Structure of α-TTP -PI(3,4)P_2_ and -PI(4,5)P_2_ complex

**Figure 1:**
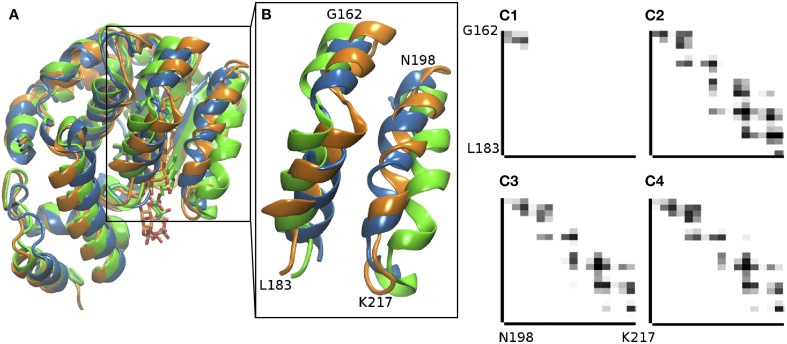
**Comparison of the α-TTP-α-Tol-PI(3,4)P_2_ complex n differentstates. (A)** the scaffold of the partially-bound structure from X-ray scattering [PDB: 3W67 (Kono et al., 2013), in green cartoon], is superimposed to the fully bound structure obtained from our MD simulations (orange cartoon) and to the closed X-ray structure with fully bound α-Tol [PDB: 1OIP (Meier et al., 2003)] (blue). **(B)** the inset report a zoom of the mobile gate helix (N198-K217) and the G162-L183 segment with which the mobile gate is in contact in closed structures. **(C)** Contact maps between the G162-L183 and the N198-K217 segments in the intermediate state captured in PDB: 3W67 (Kono et al., 2013) **(C1)**, in the α-TTP-α-Tol complex (PDB: 1OIP, Meier et al., 2003, **C2**), and in the relaxed structure from MD simulations for the α-TTP-PI(3,4)P_2_ complex **(C3)** and for the α-TTP-PI(4,5)P_2_ complex **(C4)**.

**Figure 2:**
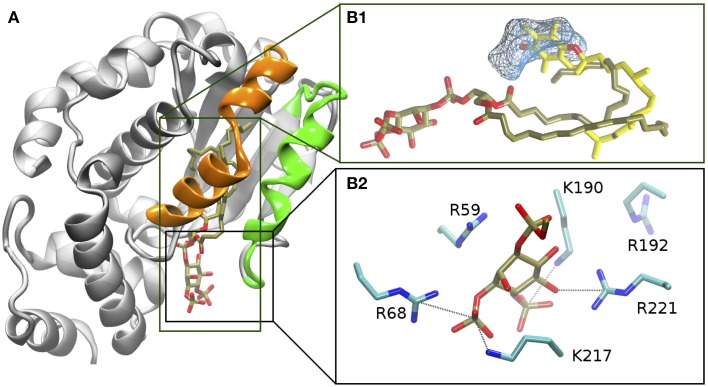
**Protein ligand Interactions in the α-TTP-PI(3,4)P_2_ complex from MD. (A)** Structure of the relaxed complex. The mobile gate and interacting region are evidenced in green and orange, respectively. **(B1)** comparison of the orientation of the fatty acid tails of PI(3,4)P_2_ after relaxation (tan licorice) with the position of α-Tol (yellow licorice) in the binding cavity from PDB: 1R5L (Meier et al., 2003). The cyan wireframe represents the area of the cavity that in our MD simulations remains occupied by water, and which corresponds to the location of the hydrophilic moiety of α-Tol. **(B2)** position of the phosphorylated head of PI(3,4)P_2_ (in tan and red licorice) and surrounding basic amio acids (in cyan and blue licorice).

The crystallographic structure of the α-TTP -PI(3,4)P_2_ complex from Arai and co-workers (Kono et al., 2013) captures an intermediate state in the plausible exchange process between α-Tol and the lipid inside the binding cavity of α-TTP. Here, we used MD simulations to model the structure of the complex in its final state by removing α-Tol and letting the structure to relax in the presence of PI(3,4)P_2_, for which a better resolved starting structure is available. Starting from the crystallographic data, during a 250 ns-long MD run, we observed penetration of the acyl chains of the lipid in the binding cavity (Figure 1A), and the mobile gate helix moved to a position in good qualitative agreement with closed-state structures previously determined for the native α-TTP-α-Tol complex (Figures 1B, 2A). The global relaxation of the structure was relatively fast, as both the sliding of the acyl chains in the binding pocket and the closure of the mobile gate occurred within the first 16 ns of MD. During the rest of the simulated time, only local adjustment of the molecular contacts were observed. The equilibrated structure recovered most of the contacts between the mobile gate and neighboring helix as in the α-TTP-α-Tol complex (Figure 1C). Difference in the contacts near the PIP_2_ head group are due to the necessity for the neighboring helices to remain slightly more open to allow accommodation of inositol in that same region.

**Figure 3:**
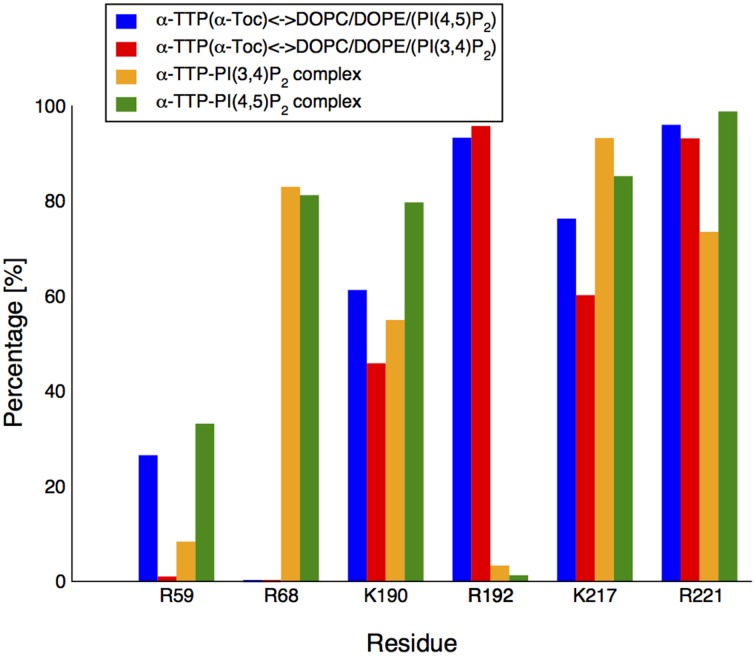
**Contact formation between the PIP's head group and basic residues in the recognition region of α-TTP**. The bars report the statistical occurrence during our MD run of salt-bridge contacts between specific side-chains of α-TTP and the head-group of the PIPs under study.

In the original X-ray image, the head group of the PI(3,4)P_2_ is clamped by the positively-charged residues at the protein's surface near the binding pocket (Figure 2B2). Such interactions were globally preserved in the closed-state complex obtained from MD. Nonetheless, both displacement of the lipid inside the cavity and mobile gate closure induced variations in the contacts between the phosphorylated head and the protein. K217 and R68 provided the most stable salt-bridge interactions with the phosphate groups. These contacts were observed for ≈ 96% and ≈ 82% of the whole simulation time, respectively (Figure 3) (percentages refer to the simulation time sampled after stabilization of the contacts between the mobile gate and the corresponding protein interface).

R221 initially interacted with the 4-phosphate, but upon relaxation of the structure, it coordinated the 5′ and 6′ OH groups, preserving stable hydrogen bonds for ≈ 73% of the simulation time. The fourth more stable interaction (observed for more than 50% of the simulation time) was provided by K190 in contact with the 4′ phosphate of the PIP head group (Figure 3).

On the contrary, R192, which is in contact with the 4′-phosphate and 5′ and 6′ hydroxyl groups in the X-ray structure, loses rapidly such interactions (they are statistically present for as low as 5% of the simulation time in the first 50 ns of the simulation).

The loss of contacts between R192 and PI(3,4)P_2_ is due to the tilting of the mobile gate upon closure of the pocket, and the corresponding conformational change observed for the side-chain of R192.

R59 is a key basic residue present in the same area, and associated to AVED mutations. Despite that, its topological position in the pocket does not allow formation of resilient contacts with PIPs. In our simulations we observed interaction with 3-phosphate only for a smaller portion of the simulated time (≈ 35%) and only for PI(4,5)P_2_. On the contrary contacts with PI(3,4)P_2_ are observed only for less than 10% of the simulation time. R59 formed instead a very stable salt-bridge with D185, present for practically the whole length of the MD run. Therefore, the importance of such residue may be rather related to the global structural stability of the N-terminal domain of α-TTP as also evidenced in former simulation by Daggett and coworkers (Bromley et al., 2013).

The hydrophobic tails of PIP in the first ns of MD form hydrophobic contacts with the apolar region of the binding cavity. In that area, the contacts between the ligand and the protein is direct, and no residual waters are observed for the whole length of the simulation. The final conformation of the fatty acid chains of PIP resemble very closely the bending of the phytyl tail of α-Tol bound to α-TTP Figure 2B1. Specifically, the stearic acid tail finds its resting position closer to the mobile gate and interacts more significantly with the residues I171, W122 and the protein segments comprising V175 to L183 and I210 to T215 belonging to the mobile gate region. The tail of the arachidonic acid chain is positioned deeper in the cavity, and interacts mainly with F158, L183, and I194.

In our simulations, PI(3,4)P_2_ did not occupy the more hydrophilic area of the binding cavity, where the chromanol ring is bound in the α-TTP-α-Tol complex. Instead, such space remained hydrated by 7 residual water molecules (Figure 2B1). Such water molecules were not fully buried inside the protein, but remained in contact with the bulk solvent through a path that passes between the protein wall and the PIP's head. Such connection of water molecules is consistent with a fully closed conformation of the protein, as a similar water channel is present also in the closed structure of the native α-TTP-α-Tol complex (Meier et al., 2003; Helbling et al., 2012).

**Figure 4:**
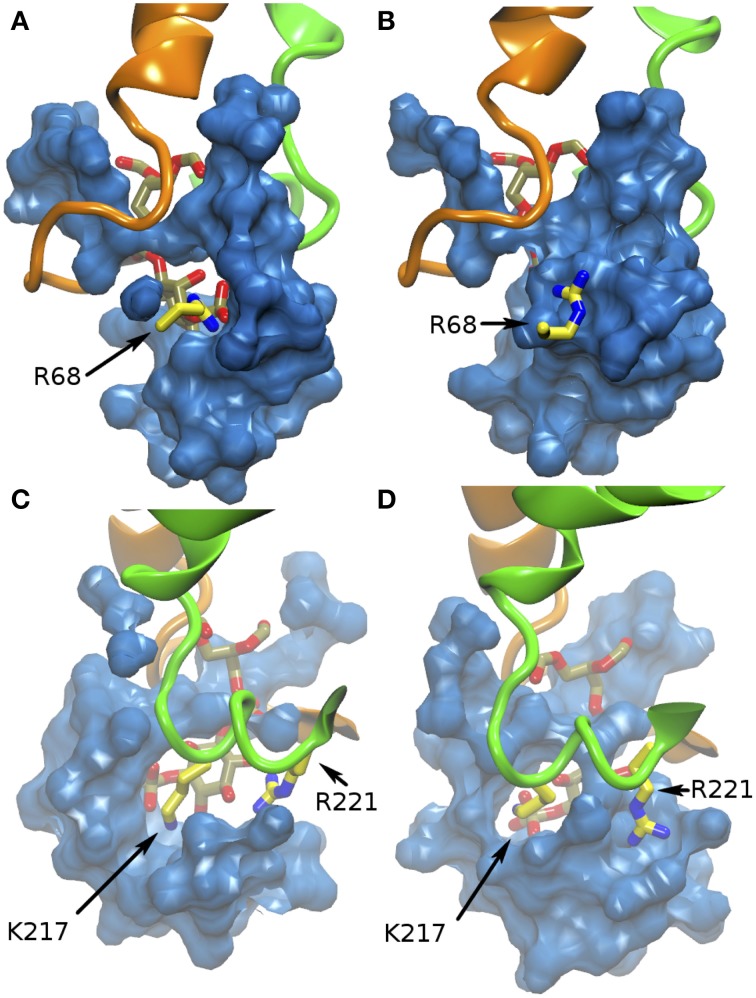
**Water interference on salt-bridges in α-TTP-PI(3,4)P_2_**. The blue surface defines the solvent occupied volume in the basic patch. **(A,B)** report two different snapshots of MD where the sale bridge between R68 and PI(3,4)P_2_ is present **(A)** or shielded by water **(B)**. **(C,D)** report for comparison the salt bridge with K217 **(C)**, which is always present during our simulation, and **(D)** a snapshot where R221, which is mostly in contact with hydroxyl groups of PI(3,4)P_2_ **(C)** is also screened by the solvent.

The water network flowing between the protein surface and the inner cavity interacts with the PIP head, and transiently disrupt salt bridges between the phosphate groups and the positively charged residues of the basic patch, particularly R221 and R68, as illustrated in Figure 4. The electrostatic interactions between PIP and K217 are instead less affected by the water dynamics (Figure 4).

Starting from the relaxed structure of the α-TTP-PI(3,4)P_2_ complex, we then modeled the binding mode of α-TTP-PI(4,5)P_2_. This second structure is of particular biological interest, since Native polyacrylamide gel electrophoresis (PAGE) analysis investigations indicate that PI(4,5)P_2_ may be the primary target of α-TTP at the plasma membrane (Kono et al., 2013).

The overall structure of the protein remained stable over more than 100 ns of MD; the RMSD of the SEC14 domain between the two complexes stabilized around a value of ≈ 0.7 Å. As for the PI(3,4)P_2_ complex, the mobile gate is well relaxed in a closed conformation, as can be seen from the contact maps reported in Figure 1C4. The different topological displacement of the phosphate groups is reflected by modifications in the strength of the hydrogen bond network between the lipid head and the residues in the basic patch of the protein (Figure 3). Specifically, the PI(4,5)P_2_ head moiety has tighter interactions with R59, K190, and R221 than PI(3,4)P_2_ (for all of them we reported up to an additional 25% of the simulation time in which we observed occurrence of the contact). In particular, R221 in this case results as the most stable contact, and practically no disruption by water is observed. Similar occurrence frequency for the salt bridge with R68 and a smaller decrease (≈ 12%) for K217 was instead reported. The neat increase of H-bonded interactions between the phosphorylated head group and the protein provides a qualitative explanation for the higher affinity of the PI(4,5)P_2_ lipid experimentally reported.

### 3.2. α-TTP-PIP_2_ complex - membrane interactions

Identification of key structural interactions between PIP and α-TTP by Arai and co-workers (Kono et al., 2013) enabled proposition of a mechanism of plasma membrane recognition and binding dominated by protein/lipid contact formation. In order to confirm and to provide a dynamical picture of such event, we simulated α-TTP binding to a model of the plasma membrane by both coarse grained (CG) and all-atom simulations.

#### 3.2.1. CG model

Our starting model contained a fully hydrated α-TTP placed at a relatively large distance (25 Å between the two closest points) from a DOPC/DOPE membrane. The membrane contained also 4 PIP_2_ molecules, in order to test the stoichiometry of the binding. Unbiased molecular dynamics simulations were then used to verify the likelihood of protein/membrane binding as suggested by Arai and co-workers (Kono et al., 2013).

**Figure 5:**
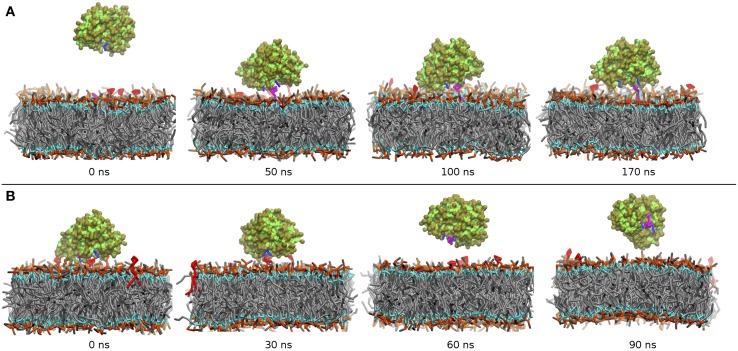
**Time series of the binding/unbinding of α-TTP to a model plasma membrane during CG MD simulations. (A)** α-TTP recognizes and binds to a PIP_2_ molecule (in purple licorice) on a mixed DOPC/DOPE/PIP_2_ lipid bilayer. **(B)** After incorporation of PIP, α-TTP detaches from the DOPC/DOPE/PIP_2_ lipid bilayer (Residues belonging to the basic patch of α-TTP are drawn in blue licorice).

The main events during our MD runs are depicted in the various panels in Figure 5A. Within the first 30 ns, the protein anchors approaches the membrane bilayer and binds to one of the PIP_2_ molecules. Such membrane anchoring occurs through interactions of the residues R192 and R221 with the phosphate groups of the PIP_2_ head moiety. These contacts are apparently very stable as, throughout the rest 140 ns of the simulation the complex between α-TTP and the lipid is never disrupted. The protein binds only to one PIP_2_ molecule, consistently with the 1:1 stoichiometry reported during *in vitro* studies (Kono et al., 2013). Within the CG model, no significant interactions between the PI(3,4)P_2_ head and R59 were observed, and the distance between the the phosphate groups of the PIP_2_ and the polar bead in the side-chain of R59 remained always greater than 6 Å. Some interactions were instead observed for PI(4,5)P_2_, with residence times only marginally weaker than in the ligand-protein complex.

Starting from the final membrane-bound complex, we then ran a second set of simulations, where the side-chains of PIP_2_ were inserted inside the binding cavity of α-TTP in agreement with the all-atom model described before. This model is representative of the protein membrane complex after the hypothetical exchange between α-Tol and PIP_2_ in the binding cavity of α-TTP has occurred.

As depicted in Figure 5B, we observed detachment of the protein from the membrane bilayer in a time scale of approximately 50 ns. After that time, the protein diffuses in the solvent and it remains fully hydrated for the rest 50 ns of the simulation. This result supports the indication that α-TTP interacts to the plasma membrane primarily through its basic patch, and that when loaded with a PIP_2_ molecule, the protein reduces significantly its affinity to the same plasma membrane.

#### 3.2.2. α-TTP-PI(4,5)P_2_-membrane system

Despite the CG simulations were able to confirm on a qualitative ground the model of lipid exchange dynamics proposed by Arai and co-workers, the same model is not appropriate to describe the atomistic detail of the protein/membrane interface. In order to both validate the findings from the CG runs, and to have an accurate description of the protein/membrane interactions, we repeated MD simulations of the α-TTP plasma membrane complex using an AA model.

The starting configuration included the fully hydrated protein located at a minimal distance of 15 Å from the membrane surface. Similarly to what observed in the CG simulations, the protein approaches the membrane and interacts with the head group of PIP_2_. In our MD simulations, binding occurs in a time-window of approximately 30 ns. Also in agreement with the CG model, we observe formation of strong salt-bridge interaction between the PIP_2_ phosphorylated head and the side-chains of residues R192 and R221. The R59 is mosty excluded from interactions with the lipid, as H-bonded contacts are observed only for a small period of the simulation time (≈ 25%). On the contrary, K190 and K217 contribute significantly (65 and 77% respectively) to the anchoring of α-TTP to PIP_2_.

**Figure 6:**
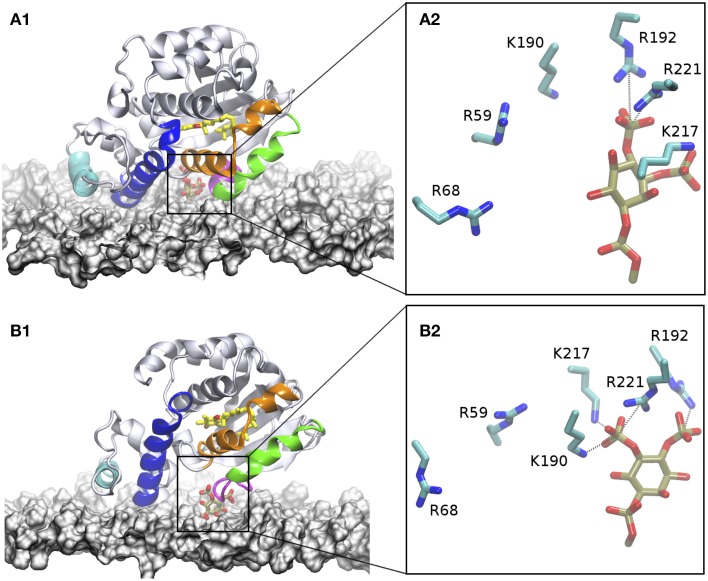
**Interaction of α-TTP with the plasma membrane**. In the figures, the membrane surface is represented by a white surface. The two N-terminal helices that interact with the membrane are highlighted in cyan and blue, the mobile gate in green, and neighboring motif in organge. the loop/short-helix motif at the C-terminal part of the mobile gate which provides both contacts to PIP_2_ and to the membrane is colored in purple. The α-Tol molecule bound to the protein is depicted in yellow licorice. the heads of PIP_2_ are also depicted in tan licorice. **(A1)** structure of the DOPC/DOPE/PtdIns(4,5)P_2_ membrane system; **(B1)** PtdIns(3,4)P_2_ membrane. **(A2,B2)** snapshots from MD highlighting the topological distribution of the residues in the basic patch of the protein around PIP_2_ when they are inserted into the membrane.

Unlike the CG model, AA simulations reveal that additional contacts at interface area between the protein and the membrane are formed after anchoring to PIP_2_. Discrepancies between the CG and AA in this region are expected due to the lack of chemical detail in the CG part needed to describe molecular recognition patterns. Specifically, we observed formation of H-bonds between the side-chains of S9, Q13, R33, R34, R68, and K219 with the polar heads of DOPC lipids. The E216 and E220 are also contributing to interactions with the amine head group of a DOPE lipid or to water mediated interactions with phosphate groups of membrane lipids. Interestingly most of these residues belong to the N-terminal domain helices as well as to the terminal part of the mobile gate (Figures 6A1,A2). That the protein-membrane interactions are localized at the N-terminal region of the protein is consistent with the experimental observation that membrane localization of Sec14 like proteins is controlled by this area of the protein (Sirokmány et al., 2006). Direct interaction with the mobile gate may be instead associated to a membrane-induced mechanism of opening of the lipid binding cavity, facilitating lipid exchange.

#### 3.2.3. α-TTP-PI(3,4)P_2_-membrane system

We repeated AA simulations where α-TTP is interacting with the plasma membrane model in the presence of α-TTP-PI(3,4)P_2_. The starting configuration of this system is the same as in the PI(4,5)P_2_ system. In our simulations, we observed the same interaction pattern as in the previous α-TTP -PI(4,5)P_2_ complex. In particular, the same residues in the basic patch of α-TTP mediate binding with the PIP's head group (Figure 6B2). In this case, R59 does not contribute to the binding. However, in this complex less stable H-bonds were formed in the case of K190 and K217. Additional differences were observed in the rest of the protein-membrane interface. In particular, the mobile gate is also in weaker contact with in contact with the membrane, as well as for the last helix of the N-terminal domain, which lays less parallel to the membrane plane (Figure 6B1). Similarly with the PI(4,5)P_2_-membrane system, most of the residues interacting with the membrane belong to the N-terminal or the mobile gate regions. Residues in the N-terminal domain comprise D64, R68, Lys71, and R75, while the basic patch region interacts with S208, K211, D216, E216, K217, and K219.

## 4. Discussion

We studied the interaction of α-TTP with PIP_2_ molecules both when they are bound into the protein's cavity or while they are embedded in the plasma membrane. In the latter case, our CG simulations agree with a 1:1 stoichiometry between α-TTP and PIP_2_ as originally proposed by Arai and coworkers (Kono et al., 2013). We observed that one single PIP_2_ molecule is sufficient to stabilize the anchoring of α-TTP to the membrane surface. Binding occurs by direct interaction of the phosphorylated head group of the lipid with the positively-charged patch at the surface of the protein near the opening of the ligand binding cavity. Such interaction appears to be very stable, as the bound lipid was never exchanged over several nanoseconds of both CG or AA simulations. We observe that the lateral lipid diffusion coefficient in CG simulations is roughly four times larger than in the all-atom run. Taking this factor as a rough estimate of the time acceleration in the CG space, we predict that α-TTP-PIP_2_ interaction at the plasma membrane can putatively live over a multi-microsecond time scale.

These long-lived electrostatic interactions between the α-TTP and PIP_2_ enabled the identification of key side chains involved in membrane recognition, interaction and possibly in the lipid exchange mechanism. Electrostatically driven protein membrane association involving PIP_2_ residues has been already observed in other systems, like for Pten tumor supressor's binding to the plasma membrane (Stansfeld et al., 2009) or for syntaxin-1A clustering to PI(4,5)P_2_ areas on the membrane (van den Bogaart et al., 2011). Mutations of most of the residues composing this portion of the protein surface are directly associated to insurgence of AVED.

When PIP_2_ is incorporated into α-TTP, we observed fast detatchment from the membrane into the bulk water, consistent with loss of the anchoring group. Our observations agree with the hypothesis that α-TTP can work by a mechanism of lipid exchange between the endosome and the plasma membrane as proposed by Arai and coworkers (Kono et al., 2013).

AA simulations clarified the fine details of the interactions of α-TTP with the PIP_2_ molecules in the two situations here investigated. Apart from the two Arginines R192 and R221 whose mutations are associated with AVED phenotypes, also amino acids R68, K190, and K217 are involved in the formation of strong salt bridge interactions with the PIP_2_ head group phosphates (Figure 3). Most importantly, in our simulations we observed that all these residues compete for interaction with the phosphate groups, with a different distribution of contacts between membrane bound or the protein bound PIP_2_.

In AA simulations of α-TTP bound to the membrane, we observed that the PIP_2_ head was not locked in one single conformation. On the contrary, there phosphate groups alternate salt-bridge interaction with several of the positively charged residues present at the protein surface.

R221 is the amino acid that most strongly interacts with PIP_2_, indicating that the observed severe AVED phenotype associated to its mutation into tryptophane may hinder plasma membrane binding. Interestingly, R59, another residue whose mutation into tryptophane is associated to insurgence of AVED, is only weakly interacting with PI(4,5)P_2_ and even less with PI(3,4)P_2_. Thus, it is likely that R59 plays a structural role for the stabilization of the helical motifs in the N-terminal region, as also evidenced by previous computational studies by Daggett and coworkers (Bromley et al., 2013). Still the clearly stronger interaction of R59 with PI(4,5)P_2_ in both the membrane and in the complex may contribute to membrane selectivity.

R68 and R192 mutations are associated to mild AVED forms. R192 is strongly interacting with the head groups of both PI(4,5)P_2_ and PI(3,4)P_2_-membrane systems. However, the same residue loses its ability to form contacts with the head group phosphates when the lipids are incorporated into the protein. A specular behavior is observed for R68, for which strong interactions are present in the PIP_2_-loaded α-TTP structures but not when PIP_2_ are embedded into the membrane (Figure 3). Such features suggest that the two residues play a role in the flipping of PIP_2_ lipids from the membrane into the pocket of the protein, shuttling the H-bond network between the protein and the phosphorylated head groups in the different conformations.

K190 and K217 are also interacting constantly with the head group in all systems investigated. The presence of a positively charged amino acid in the same structural position is conserved through evolution, as for example in CRALBP (R234) and Sec14p (R209). Also, in the case of CRALBP, the R234W mutation yields a non functional protein. The K217 is also conserved in CRALBP (K261), however in this case mutations are not directly related to unhealthy phenotypes.

Binding of to the membrane involves strong interaction of the mobile gate helix with the membrane surface. In fact, opening of the binding cavity requires shielding of a large hydrophobic area from the solvent by the same membrane. In our simulations we do not observe direct interaction of F165 and F169, which were identified as key membrane binding residues in previous studies (Zhang et al., 2011). Nonetheless, both these amino acids reside in a key loop region that we demonstrated to undergo large conformational changes to facilitate the displacement of the mobile gate (Helbling et al., 2014). Thus, the topological position of the membrane-bound α-TTP suggests that interaction of F165 and F169 with the membrane may be crucial to facilitate the opening of the binding cavity. We remark that in both α-TTP-membrane systems we studied, we did not observed any changes in the binding pocket where α-Tol is located. Thus, rearrangement of the residues at the protein-membrane interface and the basic patch during the recognition-anchoring of the α-TTP is not affecting ligand binding. Therefore, we may assume that deeper penetration of the mobile gate region into the hydrophobic area of the membrane is needed to activate the extraction of α-Tol. The understanding of the detailed molecular mechanisms for the lipid exchange process may be addressed in future studies specifically dedicated to this problem.

Finally, the N-terminal domain of Sec14 like proteins has also a direct role in membrane recognition and binding, as evidenced by studies on Sec14 like protein chimeras by Arai and coworkers (Horiguchi et al., 2003). During the protein-membrane interaction a distortion of the N-terminal domain toward the membrane plane is observed. Such conformational changes are induced by extended interactions between the side-chains of residues forming the N-terminal helices and the membrane plane. Nonetheless, our simulation times were too short to be able to determine any direct structural correlation between results from Horiguchi et al. (2003) and our observations.

## Concluding remarks

The mechanism of recognition and interaction of α-TTP with the plasma membrane have been described by means of multiscale MD. CG simulations were capable of providing a good qualitative picture of the lipid recognition and binding interface. Atomistic simulations were used to obtain high-resolution insights on the interactions of α-TTP with different PIP_2_ both while inserted in the plasma membrane and while bound to the protein. Globally, our studies confirmed the model of lipid exchange recently proposed by Arai and coworkers (Kono et al., 2013), and highlighted the role of several amino acids associated to insurgence of both serious and mild forms AVED. Our study underlined the relevance of conformational dynamics at the protein/membrane interface, and set a starting point for future computational investigations on the lipid exchange mechanism at atomistic models of the plasma and endosomal membranes.

## Funding

AS thanks the Swiss National Science Foundation for funding through the grant n. 31003A_156419. MC acknowledges the support of the Norwegian Research Council through the CoE Centre for Theoretical and Computational Chemistry (CTCC) Grant Nos. 179568/V30 and 171185/V30.

### Conflict of interest statement

The authors declare that the research was conducted in the absence of any commercial or financial relationships that could be construed as a potential conflict of interest.
